# Src signaling in a low-complexity unicellular kinome

**DOI:** 10.1038/s41598-018-23721-8

**Published:** 2018-03-29

**Authors:** Hiroshi Suga, W. Todd Miller

**Affiliations:** 1 Faculty of Life and Environmental Sciences, Prefectural University of Hiroshima, 727-0023 Shobara, Hiroshima Japan; 2 Department of Physiology and Biophysics, Stony Brook University, Stony Brook, NY 11794 USA

## Abstract

*Creolimax fragrantissima* is a member of the ichthyosporean clade, the earliest branching holozoan lineage. The kinome of *Creolimax* is markedly reduced as compared to those of metazoans. In particular, *Creolimax* possesses a single non-receptor tyrosine kinase: CfrSrc, the homolog of c-Src kinase. CfrSrc is an active tyrosine kinase, and it is expressed throughout the lifecycle of *Creolimax*. In animal cells, the regulatory mechanism for Src involves tyrosine phosphorylation at a C-terminal site by Csk kinase. The lack of Csk in *Creolimax* suggests that a different mode of negative regulation must exist for CfrSrc. We demonstrate that CfrPTP-3, one of the 7 tyrosine-specific phosphatases (PTPs) in *Creolimax*, suppresses CfrSrc activity *in vitro* and *in vivo*. Transcript levels of CfrPTP-3 and two other PTPs are significantly higher than that of CfrSrc in the motile amoeboid and sessile multinucleate stages of the *Creolimax* life cycle. Thus, in the context of a highly reduced kinome, a pre-existing PTP may have been co-opted for the role of Src regulation. *Creolimax* represents a unique model system to study the adaptation of tyrosine kinase signaling and regulatory mechanisms.

## Introduction

While Ser/Thr phosphorylation is used as a signaling mechanism in all branches of the eukaryotes, the presence of the phosphotyrosine-based signaling system is limited to some specific subtrees, including that of metazoans, indicating that it arose relatively recently in evolutionary history^1,2^. In metazoans, pTyr-based signaling plays essential roles in cell-to-cell communication and in the regulation of cell proliferation and differentiation^1^. The components of the pTyr signaling machinery (tyrosine kinases, tyrosine phosphatases, and pTyr-binding domains) have also been discovered in several groups of premetazoans, indicating that the evolution of pTyr-based signaling predated the advent of multicellularity^3–5^.

The human genome encodes 90 tyrosine kinases, which are divided into two groups: the 58 transmembrane receptor tyrosine kinases (RTKs) and the 32 cytoplasmic or nonreceptor tyrosine kinases (NRTKs)^6,7^. The kinase catalytic domains of these two groups are closely related to each other, while the remainders of the proteins are divergent and typically contain additional modular signaling domains. The extracellular domains of the RTKs are particularly diverse, as expected, because these portions of the RTKs contain the sequences necessary for specific binding of their ligands^8^.

In humans, many families of NRTKs (the Src, Abl, Tec, Csk, and Frk families) possess a core architecture that consists of SH3, SH2, and kinase catalytic domains^9,10^. This arrangement of domains is important for proper enzyme regulation, and for recognition of cellular substrates^11–13^. This core architecture is present in the kinomes of unicellular filastereans and choanoflagellates, indicating that it was already established before the divergence of filastereans from choanoflagellates and metazoans^3–5,9^. Evolution of the SH2-catalytic domain combination has been postulated to contribute to the development of positive and negative feedback loops found in metazoan signaling^2^. There are eight Src family kinases (SFKs) in humans. Three of them (Src, Fyn, and Yes) are expressed in most tissues, while the others (Blk, Fgr, Hck, Lck, and Lyn) are more selectively expressed in particular tissues^12^. SFKs are involved in the development and differentiation of specific cell types in mammals^14^. In many cell types, however, the presence of multiple SFKs (and other related NRTKs) complicates the analysis of signal transduction, as they can have redundant functions. Humans also have approximately 100 protein tyrosine phosphatase family genes, of which 37 encode tyrosine-specific phosphatases (PTPs). The others are dual specificity phosphatases, which can dephosphorylate both pSer/pThr and pTyr^2,15^.

**Figure 1:**
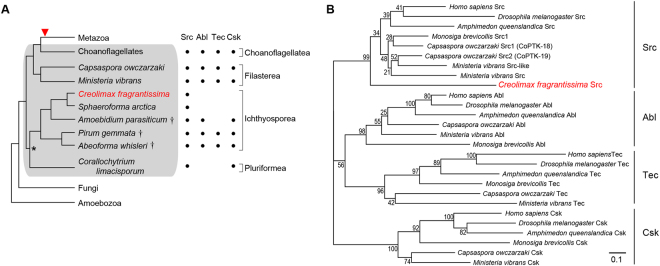
(**A**) Phylogeny of eukaryotes leading to metazoans. The relationship between classes is schematically depicted based on Torruella *et al*.^17^. *A relationship where an inconsistency was noted by Hehenberger *et al*.^16^. Unicellular holozoans are shaded in grey. Red triangle depicts the origin of animal multicellularity. Taxonomic classes are shown on the right. The table indicates the presence (filled circle) and absence of the four NRTKs, identified by mining the whole genomic and RNAseq data or solely RNAseq data^†^. (**B**) Phylogenetic tree of NRTKs. The amino acid sequences of kinase catalytic domains, SH2 domains, and SH3 domains were used for the inference. Only the Src-type NRTKs with SH3-SH2-kinase architecture from selected animal lineages, *M*. *brevicollis*, two filastereans, and *Creolimax* are included. Bootstrap values from 200 times inference replicates are shown at branches. The *Creolimax* CfrSrc is shown in red. Scale bar, 0.1 substitution/site.

*Creolimax fragrantissima* (*Creolimax* hereafter) is a member of the ichthyosporean clade, the earliest branching holozoan lineage (Fig. 1A)^16,17^. The lifecycle of *Creolimax* consists of at least three stages^4^. In the initial growth stage, a small uninucleate cell develops multiple nuclei in its cytoplasm. In the second maturation stage, a dynamic rearrangement of cytoplasm occurs and all nuclei cellularize in a relatively short time (~2 hours). The motile amoebae that are produced are released through tears in the mother cell wall and migrate on a substrate in the third stage. The regulatory signals that trigger these transitions between cell types are unknown. The tyrosine kinome of *Creolimax* is smaller than that of filastereans, choanoflagellates, and metazoans, with 31 total TKs^18^. Strikingly, *Creolimax* possesses only a single NRTK: the Src homolog (CfrSrc). The genome of *Creolimax* also encodes only 7 tyrosine-specific phosphatases (PTPs), while other ichthyosporean lineages such as *Abeoforma whisleri*, *Pirum gemmata*, and *Amoebidium parasiticum*, and the independent holozoan lineage *Corallochytrium limacisporum* possesses richer complements of NRTKs and PTPs^18^. Thus, studies of tyrosine kinase signaling in *Creolimax* can shed light on kinase function in the context of a simplified repertoire of intracellular signaling components.

In this study, we compare and contrast *Creolimax* CfrSrc with metazoan SFKs. Similarly to c-Src, purified CfrSrc shows robust tyrosine kinase activity and is regulated by autophosphorylation. Interestingly, the enzyme possesses a leucine residue in the “gatekeeper” position, rendering it resistant to pharmacological ATP-competitive inhibitors; in this respect it resembles mutant forms of tyrosine kinases that arise in patients treated with these drugs. We also cloned and expressed the *Creolimax* tyrosine phosphatases, and demonstrate that one (CfrPTP-3) has high activity against CfrSrc. RNAseq data revealed that expression of CfrPTP-3 is over 5 times higher than that of CfrSrc during the multinucleate stage, and in the amoeboid stage the expression of CfrPTP-3 nearly doubles. We analyzed the role of CfrSrc *in vivo*. *Creolimax* cells overexpressing CfrSrc show growth defects and never reach the maturation stage to produce amoebae. Co-expression of CfrPTP-3 rescues these phenotypes, suggesting that an existing PTP was co-opted for the role of Src regulation in the highly reduced kinome of *Creolimax*.

## Results

### Properties of Creolimax CfrSrc

We performed a phylogenetic analysis to study the relationship between *Creolimax* CfrSrc, the sole NRTK in this species, and the NRTK families containing the core SH3-SH2-kinase architecture (Src, Abl, Tec, and Csk) (Fig. 1B). This analysis supported the classification of CfrSrc as an SFK with a bootstrap value of 99%. The genomes of both the choanoflagellate *M*. *brevicollis* and the filastereans *C*. *owczarzaki* and *Ministeria vibrans* contain at least one gene in each of the four NRTK families, Src, Abl, Tec, and Csk (Fig. 1A). Moreover, the transcriptome data from the ichthyosporean *A*. *parasiticum* revealed NRTK genes belonging to each of the NRTK families except the Tec family^18^. In contrast, the genome of *Sphaeroforma arctica*, a closely-related ichthyosporean species to *Creolimax*, contains only one NRTK gene (Src), similar to *Creolimax*. These data indicate that a drastic reduction of the NRTK repertoire occurred relatively recently in the common ancestor of *Creolimax* and *S*. *arctica*.

**Figure 2:**
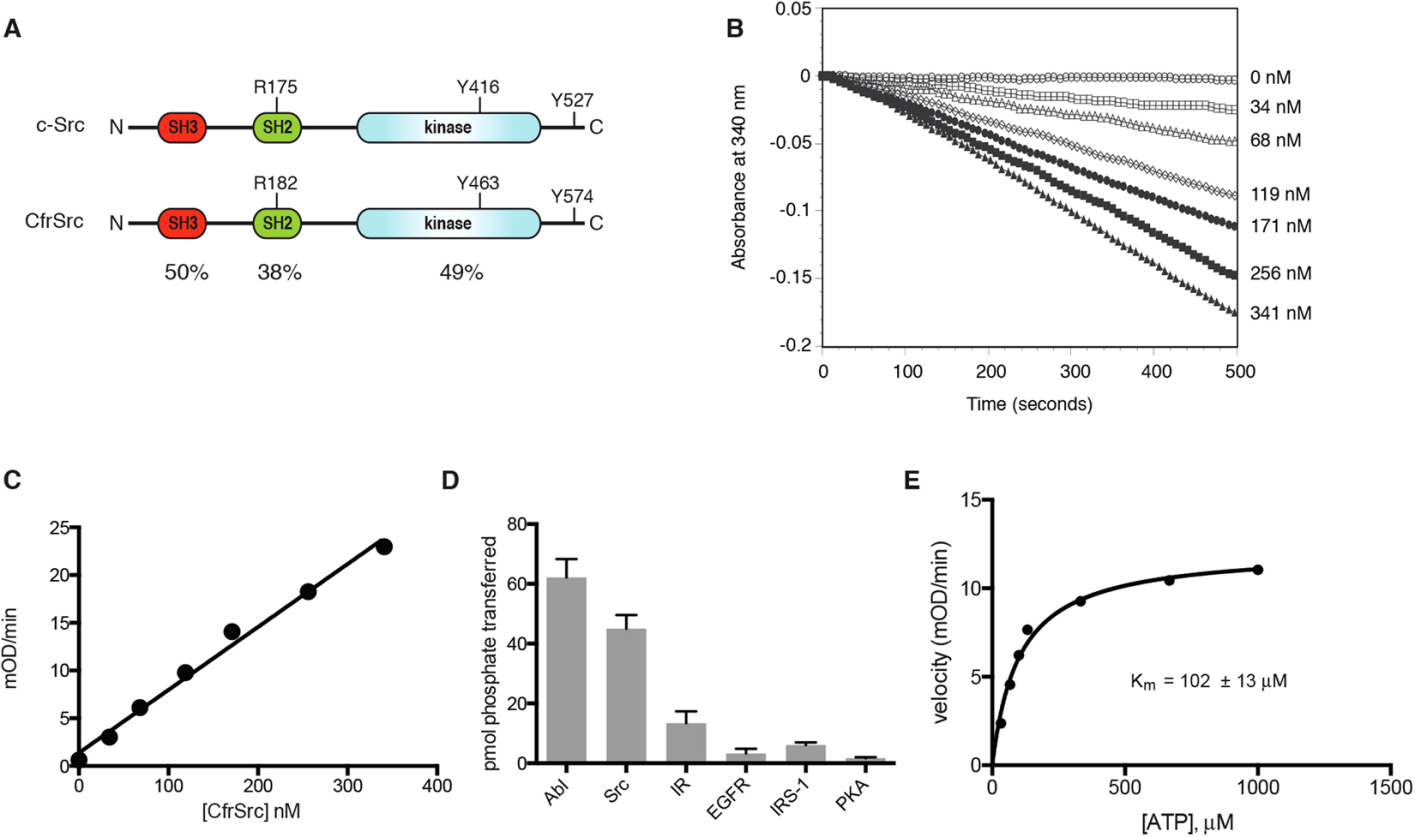
Characterization of CfrSrc activity. (**A**) Comparison of the domain structures of CfrSrc and human c-Src. Key residues are indicated, and the percentages of amino acid identity are shown beneath each domain. (**B**) The activity of purified CfrSrc toward the Src synthetic peptide was measured using the continuous spectrophotometric assay. Absorbance data were recorded every 8 seconds. Concentrations of CfrSrc are shown to the right of the graph. This experiment was repeated 3 times with similar results, and a representative graph is shown. The initial rates are replotted against [enzyme] in panel (**C).** (**D**) The activity of purified CfrSrc toward various peptide substrates was measured in duplicate using phosphocellulose paper binding assays with [γ-^32^P]ATP. The peptide sequences were: Abl peptide, EAIYAAPFAKKKG; Src peptide, AEEEEIYGEFEAKKKKG; IR peptide, KKEEEEYMMMM; EGFR peptide, AEEEEYFELVAKKKG; IRS-1 peptide, KKSRGDYMTMQIG; and PKA peptide, LRRASLG. Values are +/−S.D. (**E**) Initial rates were measured at various concentrations of Src peptide using the continuous assay. The rates were replotted against substrate concentration and fit to the Michaelis-Menten equation.

We cloned the cDNA encoding full-length CfrSrc (residues 1–586) from *Creolimax*. CfrSrc has the SH3-SH2-kinase domain architecture that is conserved in all Src family kinases, from other unicellular organisms (choanoflagellate, filasterean) to human (Figs 2A and S1). Key Src functional residues are present in CfrSrc. The SH2 domain has an Arg residue (R182) within the sequence YLVRES, which is predicted to interact with pTyr-containing ligands^19^. A tyrosine residue (Y463) is present within the predicted activation loop (A-loop); in other SFKs, this tyrosine is the major site for autophosphorylation, which enhances catalytic activity^20^. Another important regulatory tyrosine in Src family kinases is Y527 in the C-terminal tail (chicken c-Src numbering). In mammals, Y527 is phosphorylated by Csk kinase, leading to an intramolecular interaction with the SH2 domain and inhibition of kinase activity^19,21,22^. CfrSrc possesses a tyrosine residue at the C-terminus (Y574), but *Creolimax* lacks Csk kinase, suggesting a different mechanism of regulation.

Within the kinase catalytic domain, there are significant differences between the sequence of CfrSrc and those of mammalian SFKs. CfrSrc possesses a large insertion near the hinge that separates the N- and C-terminal lobes of the kinase domains (Fig. S1); this is similar to the insertion seen in several metazoan RTKs. The phosphate-binding loop (P-loop) and A-loop sequences of CfrSrc are also divergent from those of other SFKs. The A-loop has an insertion between the kinase-conserved DFG motif and the predicted autophosphorylation site. From the sequence alone, it was not clear whether these changes in the catalytic domain would affect kinase activity.

We expressed His-tagged CfrSrc in Sf9 insect cells and purified the kinase by immobilized metal affinity chromatography (IMAC) using nickel resin. The purified enzyme had robust activity towards tyrosine-containing synthetic peptides, and showed the expected linear relationship between velocity and enzyme concentration (Fig. 2B,C). We tested enzymatic activity against a series of synthetic peptides containing recognition motifs for various kinases. The highest activity in this experiment was measured with an Abl kinase substrate, although there was also good activity with a Src peptide (Fig. 2D). The enzyme’s activity towards receptor tyrosine kinase substrates was relatively low, and there was only background activity towards Kemptide, a substrate for the Ser/Thr kinase PKA. Using the Src peptide, we measured an apparent K_m_(ATP) of approximately 100 μM for CfrSrc (Fig. 2E).

The SH2 domain of mammalian c-Src is involved in intra- and inter-molecular interactions that govern the activity and specificity of the kinase^11,19,21,23^. These interactions depend on the ability of the SH2 domain to bind phosphotyrosine-containing peptide sequences. To test whether this ability is present in *Creolimax* Src, we cloned and expressed the isolated SH2 domain as a fusion protein with GST. We used fluorescence polarization to test for an interaction between the purified SH2 domain and a synthetic peptide containing pYEEI, the optimal sequence for binding to the SH2 domain of c-Src^24^. These experiments showed that the SH2 domain is functional, and could bind pYEEI with a K_d_ of 2.7 μM (Fig. S2). This is higher than the value of 200 nM reported for the pYEEI peptide binding to the isolated SH2 domain of mammalian c-Src^25^. It is also higher than the K_d_ value of 140 nM measured for the binding of pYEEI to the SH2 domain of MbSrc1 from the unicellular choanoflagellate *M*. *brevicollis*^26^. Thus, it appears that the SH2 domain of CfrSrc has a lower affinity for pTyr-containing sequences than other Src family kinases (including other unicellular SFKs); additionally or alternatively, it may possess a divergent specificity for amino acids surrounding pTyr. While the key pTyr-binding residues are conserved in the CfrSrc SH2 domain, a *Creolimax*-specific 7-residue insertion could potentially confer the altered binding (Fig. S1).

### Gatekeeper sequence of CfrSrc

**Figure 3:**
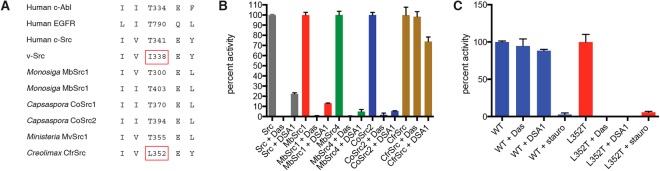
The gatekeeper position of CfrSrc. (**A**) Comparison of sequences surrounding the gatekeeper residues of various tyrosine kinases. Most kinases have Thr at this position; exceptions are viral Src and CfrSrc. (**B**) Human c-Src, *Monosiga brevicollis* MbSrc1 and MbSrc4, *Capsaspora owczarzaki* CoSrc2, and *Creolimax* CfrSrc (400 nM) were assayed in duplicate in the absence or presence of 5 μM dasatinib or DSA1 using the phosphocellulose binding assay. Values are +/−S.D. (**C**) Purified wild-type or L352T CfrSrc (400 nM) were assayed in duplicate in the absence or presence of 5 μM dasatinib, DSA1, or staurosporine. Values are +/−S.D. Experiments in this figure were repeated 3 times with similar results.

Many small-molecule ATP-competitive protein kinase inhibitors bind into a hydrophobic pocket deep in the active site cleft. A conserved threonine residue (the ‘gatekeeper’ residue) controls access to this pocket, and mutation of this threonine with bulkier residues is a common mechanism for clinical resistance to kinase inhibitors^27,28^. Clinically relevant tyrosine kinases contain Thr at the gatekeeper position, as do unicellular SFKs from *M*. *brevicollis*, *C*. *owczarzaki*, and *M*. *vibrans* (Fig. 3A). v-Src, the activated form of Src isolated from Rous sarcoma virus, contains a bulky isoleucine at the gatekeeper position (Fig. 3A). Homology modeling of CfrSrc shows that a leucine residue (L352) is in the gatekeeper position in CfrSrc (Figs 3A and S4). This observation suggested that CfrSrc might be resistant to small-molecule pharmacological Src inhibitors. Indeed, while human c-Src, MbSrc1 and MbSrc4 from *M*. *brevicollis*, and CoSrc2 from *C*. *owczarzaki* were all strongly inhibited by the clinical Src inhibitor dasatinib, CfrSrc was completely resistant to this drug (Fig. 3B). Also, in contrast to the other forms of Src tested, CfrSrc was only weakly (ca. 30%) inhibited by DSA1, a pyridinyl triazine compound that binds to the DFG-flipped conformation of c-Src and inhibits the T338I gatekeeper mutant form of c-Src^29^ (Fig. 3B). CfrSrc is inhibited by staurosporine, an ATP-competitive inhibitor that binds to many kinases with high affinity, but with low selectivity (Fig. 3C). To test whether the lack of dasatinib inhibition was attributable to the presence of L352 in the gatekeeper position, we produced a L352T mutant form of CfrSrc in Sf9 insect cells. The purified L352T mutant was strongly inhibited by both dasatinib and DSA1 (Fig. 3C). The IC_50_ value for dasatinib for the L352T mutant was approximately 11 nM. These results show that the wild-type form of CfrSrc resembles mutant forms of SFKs that arise via resistance to pharmacological ATP-competitive inhibitors.

### Phosphorylation of CfrSrc

Src-family kinases are regulated by intermolecular autophosphorylation on the A-loop. This modification destabilizes the autoinhibited conformation, stabilizes the active conformation, and leads to enzyme activation. This mode of regulation is present in SFKs from unicellular choanoflagellates and filastereans^30–33^. We dephosphorylated purified CfrSrc using YOP tyrosine phosphatase and showed that activity was reduced to background levels (Fig. S3A). The reduction in activity was associated with a decrease in overall tyrosine phosphorylation, as measured by anti-pTyr Western blotting (Fig. S3B). After removing YOP, we incubated the dephosphorylated sample with ATP, which led to a large increase in CfrSrc activity (Fig. S3A) and an increase in tyrosine phosphorylation (Fig. S3B). These results indicate that CfrSrc is also positively regulated by autophosphorylation.

CfrSrc is the only NRTK present in *Creolimax*; Csk, which inactivates Src in metazoans, is missing. Thus, CfrSrc activity should be regulated by other mechanisms than the phosphorylation by Csk. However, given the relatively recent loss of Csk in the course of ichthyosporean evolution, the possibility that CfrSrc could still be regulated by Csks from other holozoans remains. Therefore we tested whether recombinant Csk could inhibit CfrSrc. We incubated CfrSrc with human Csk (hCsk) or MbCsk from *M*. *brevicollis*, then assayed CfrSrc activity towards a synthetic peptide substrate with the radioactive [γ-^32^P]ATP assay. (Under similar *in vitro* conditions, hCsk inhibits the activity of human c-Src, but not *M*. *brevicollis* Src)^26^. The activity of CfrSrc was not inhibited in these experiments. Instead, we observed a small but reproducible increase in CfrSrc activity (Fig. S3C). We obtained similar modest increases for the filasterean SFKs CoSrc1 and CoSrc2 (from *C*. *owczarzaki*), and MvSrc1 and MvSrc2 (from *M*. *vibrans*, another filasterean lineage)^31,33^. These increases may be due to complex formation between the two enzymes, which could stabilize the open, active form of CfrSrc. We tested whether hCsk or MbCsk could phosphorylate CfrSrc by anti-pTyr Western blotting (Fig. S3D). In this experiment, we suppressed CfrSrc autophosphorylation by using the L352T mutant form of CfrSrc in the presence of the inhibitor DSA1. DSA1 blocks the activity of L352T (Fig. 3C), but this class of inhibitors is inactive against Csk^34^. Human Csk phosphorylated CfrSrc L352T weakly, as compared with a control L352T autophosphorylation reaction in the absence of DSA1 (Fig. S3D). MbCsk did not display any activity towards CfrSrc.

### Analysis of Creolimax PTPs

**Figure 4:**
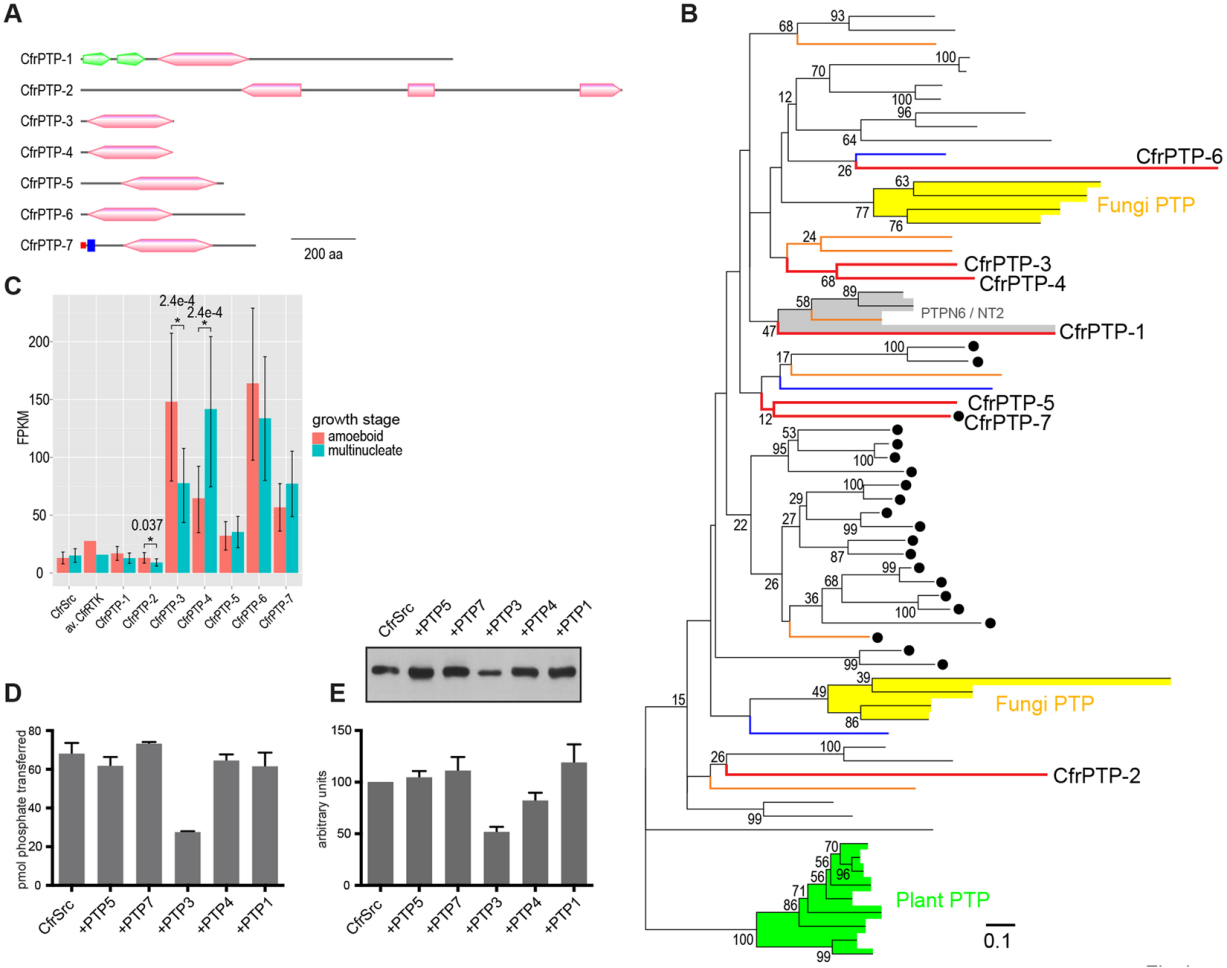
*Creolimax* tyrosine phosphatases. (**A**) Domain diagrams of the seven *Creolimax* tyrosine phosphatases. The PTP domain is shown in pink. The SH2 domain, signal peptide, and transmembrane segment are shown in green, red, and blue, respectively. (**B**) Phylogenetic tree of PTPs. Bootstrap values from 500 times inference replicates by the Rapid Bootstrapping Algorithm^56^ are shown, except those less than 10%. PTPs from metazoans, *C*. *owczarzaki*, *Creolimax*, and *Dictyostelium discoideum* are shown in black, orange, red, and blue lines, respectively. PTPs from fungi and plants are shaded yellow and green, respectively. The sole pan-holozoan PTP family (PTPN6 family or NT2 subtype) is shaded grey. Receptor PTPs are marked by filled circles. Scale bar, 0.1 substitution/site. (**C**) RNAseq expression analysis. The FPKM values for CfrSrc and 7 CfrPTPs, together with the average value of the 30 receptor TKs, are shown. Red and blue bars indicate the expression in the amoeboid- and multinucleate-stage cells, respectively. The error bar indicates the 95% confidence intervals. Significant stage-specific differences are marked by asterisks, together with the FDR-adjusted p-value. (**D**) Purified CfrSrc was incubated with immobilized CfrPTPs, then assayed in duplicate for kinase activity toward the Src peptide using the phosphocellulose binding assay. A representative from 3 independent experiments is shown +/−S.D. (**E**) After incubation with CfrPTPs, Src autophosphorylation was measured by Western blotting with anti-pTyr antibodies (top). Results from 4 separate experiments were quantified (bottom) and are shown +/−S.D. The 4 full-length blots are shown in Supplemental Figure 6.

Our results with YOP phosphatase (Fig. S3A) demonstrate the potential of PTPs to regulate the activity of CfrSrc. The genome of *Creolimax* contains 7 predicted PTPs (Fig. 4A). A phylogenetic analysis (Fig. 4B) of the catalytic domain revealed a complex evolutionary history of PTPs. Unlike TKs, PTPs are found in plants and fungi, suggesting an earlier origin of PTPs than TKs. While the plant PTPs are monophyletic, fungal PTPs are separated into two clusters that show shallow branchings from the holozoan PTPs. This indicates that the closest common ancestor of opisthokonts (holozoans + fungi and their closely-related protists) already had a reasonably divergent complement of PTPs, which was reduced in fungi after the separation from holozoans. However, the *Creolimax* PTPs are not closely related either to fungal PTPs or to plant PTPs, or even to any metazoan PTP family, except CfrPTP-1, which belongs to the PTPN6 family (NT2 subtype) having SH2 domains in common at the N-terminus. This is in sharp contrast to the filasterean *C*. *owczarzaki*. Similar to *Creolimax*, *C*. *owczarzaki* has only 7 predicted PTPs, of which 6 genes are putative homologs to specific PTP families^5^. It is possible that *Creolimax* attuned its PTP complement to the extremely reduced kinome.

The RNAseq expression analysis revealed relatively high levels of CfrPTPs compared to that of CfrSrc (Fig. 4C). The expression of CfrPTP-3, CfrPTP-4, and CfrPTP6 are particularly high, 5.0–12.7 times higher than CfrSrc in the motile amoeboid stage and 5.2–9.4 times higher in the sessile multinucleate growth stage. The averaged expression level of 30 *Creolimax* RTKs is comparable (2.1 times and 1.0 times higher in the amoeboid stage and the multinucleate stage, respectively) to the expression of CfrSrc. The expression of three CfrPTPs (CfrPTP-2, CfrPTP-3, and CfrPTP-4) differs statistically significantly between the two developmental stages. These results raise the possibility that the activity of CfrSrc is regulated by certain CfrPTPs in a stage-specific manner.

Two of the *Creolimax* PTPs, CfrPTP-2 and CfrPTP-6, have unusual insertions in the catalytic domain and/or diverged amino acid sequences in the catalytic center and other parts of the sequences, as indicated also by their long branches in the phylogenetic tree. Thus, they are predicted to be inactive as phosphatases. To test for regulation of CfrSrc, we cloned, expressed, and purified the remaining five PTPs (CfrPTP-1, CfrPTP-3, CfrPTP-4, CfrPTP-5, and CfrPTP-7) (Fig. S5A). For CfrPTP-3, CfrPTP-4, and CfrPTP-5, we expressed the entire genes. For CfrPTP-1, we removed the large region C-terminal to the PTP domain. For CfrPTP-7, we expressed a form lacking the predicted N-terminal transmembrane sequence. First, we confirmed that all of the PTPs were active towards the synthetic substrate p-nitrophenol phosphate (PNPP): incubation of 2 μg PTP with 10 μmol PNPP for 5 minutes gave 0.024 μmol, 0.062 μmol, 0.062 μmol, 0.037 μmol, and 0.025 μmol p-nitrophenol for CfrPTP-1, CfrPTP-3, CfrPTP-4, CfrPTP-5, and CfrPTP-7, respectively. Next, we incubated the purified, immobilized PTPs with CfrSrc, then removed the PTPs by centrifugation. We assayed CfrSrc activity towards a synthetic peptide substrate using the radioactive kinase assay. Only CfrPTP-3 gave significant (≈60%) inhibition of CfrSrc activity (Fig. 4D). We carried out similar experiments to test for effects on CfrSrc autophosphorylation, as measured by anti-pTyr Western blotting. CfrPTP-3 was also the most potent inhibitor of autophosphorylation (≈50% inhibition), while in this assay CfrPTP-4 also showed a smaller degree of inhibition (Fig. 4E). We characterized the activity of CfrPTP-3 further using the Malachite green assay. We confirmed that CfrPTP-3 is active towards phosphotyrosine-containing synthetic peptides, as well as towards pTyr itself, while it has no activity towards pSer (Fig. S5B and C).

### Functional analysis of CfrSrc and CfrPTP-3 *in vivo*

To examine the activity of CfrSrc and CfrPTP-3 in *Creolimax* cells, we made overexpression constructs with the beta-tubulin promoter and transfected them into size-fractionated cells (≤10 μm in diameter) by electroporation. All experiments were performed with a marker construct that drives Venus expression, and transformants were selected under a fluorescent microscope. In the Venus-expressing transformants, the co-transfected constructs are expected to drive the target gene proportionally to the fluorescent signal^4^.

**Figure 5:**
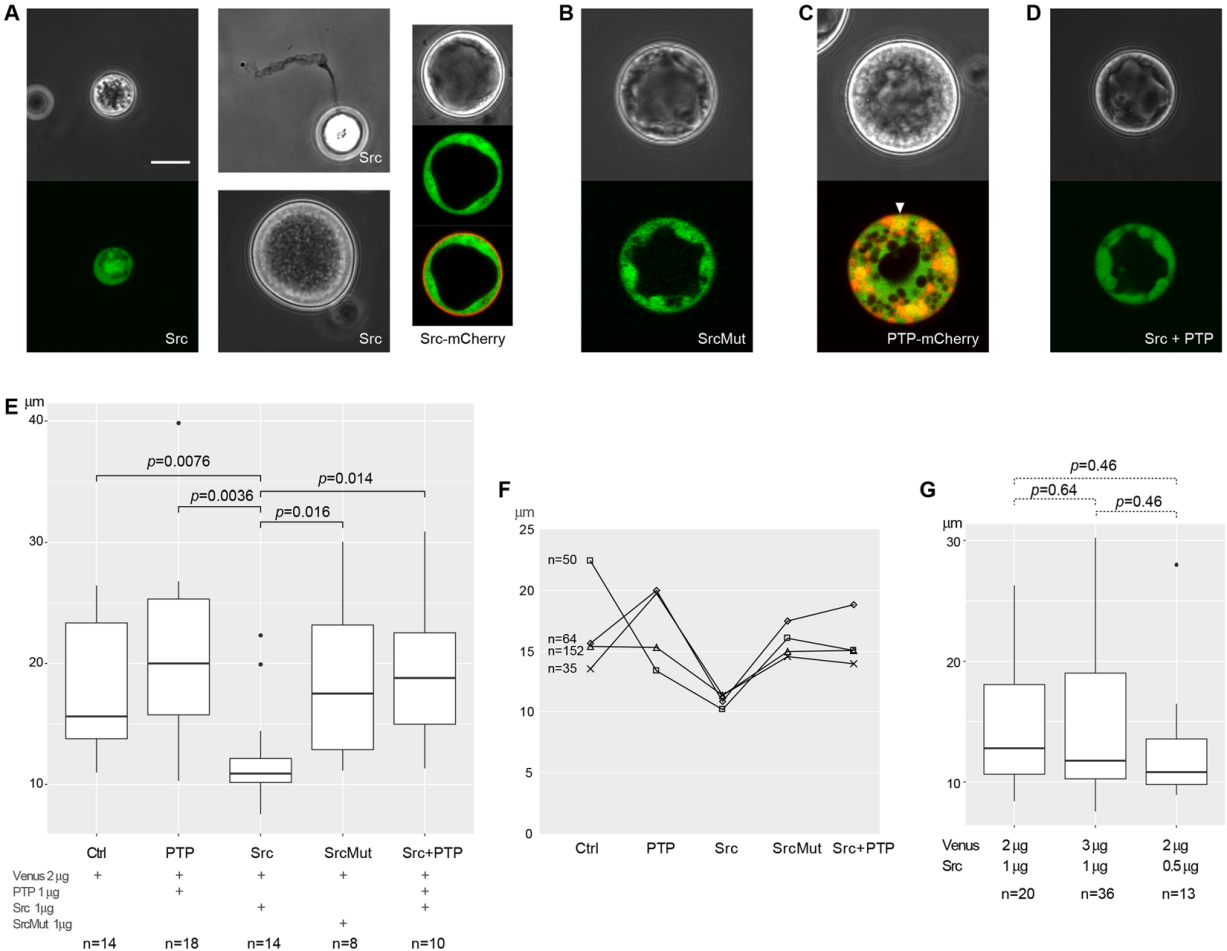
*In vivo* functional analyses of CfrSrc and CfrPTP-3. The expression of CfrSrc or CfrSrc-mCherry (**A**), CfrSrcMut (K308M and Y574F) (**B**), CfrPTP-3-mCherry (**C**), and CfrSrc + CfrPTP-3 (**D**) were overdriven by the beta-tubulin promoter of *Creolimax*. The Venus-expressing construct was always co-transfected to screen the transformants (pseudocolored in green). In the right panel and the middle-lower panel in (**A**), the rare transformants that grew even with excessive CfrSrc are shown. In the right panel in (**A**), CfrSrc is fused with mCherry to localize the protein by red fluorescence. The arrowhead in (**C**) indicates one of the nuclei, in which both Venus and mCherry signals are present. (**E**) Diameters of transformants obtained in an experiment starting from the same culture. The amount of constructs in 10 μl of electroporation buffer and the number of transformants are shown on the bottom. The p-values of Wilcoxon test with Benjamini-Hochberg correction are shown only for statistically significant pairs. (**F**) The experiment was repeated for 4 times with 4 independent cultures and the median values of transformant diameters are plotted with the total number of obtained transformants. (**G**) The growth suppression activity of CfrSrc was examined both with a reduced ratio of CfrSrc construct (middle box plot) and with a half amount of CfrSrc (right), compared with the standard condition (left). The p-values of Wilcoxon test with Benjamini-Hochberg correction are shown above the plots. Scale bar, 10 μm.

*Creolimax* cells showed a strong growth defect phenotype with the overexpressed CfrSrc (Fig. 5A left), with the average diameter remaining ~10 μm (normally 15–20 μm) 2 days after transformation. In most cases the transformants died without growth. A single large vacuole, which is usually observed in the center of a healthy *Creolimax* cell^4,35^, was rarely seen. Most transformants were completely filled with cytoplasm. We frequently observed the “smoking” phenotype, in which the cytoplasm with some insoluble materials is pushed out of a crack in the cell wall (Fig. 5A top-middle). A few transformants managed to escape from the growth retardation effect and grew larger (Fig. 5A bottom-middle), although the cells were usually not completely round, and never reached the maturation stage to produce amoebae. When fused to mCherry, CfrSrc was observed at the cell surface (Fig. 5A right), suggesting that the protein is properly localized, bound to the cell membrane by N-terminal myristoylation.

Next, we mutated the CfrSrc gene both at the ATP-binding site and at the autophosphorylation site (Fig. S1), converting the protein into a non-functional form (CfrSrcMut). When the CfrSrcMut was overexpressed, no growth defect was observed compared to the control cells, in which only the marker Venus gene was expressed (Fig. 5B). This indicates that the tyrosine phosphorylation activity of CfrSrc accounted for the growth defect observed in Fig. 5A, excluding the possibility that a structural change in the cell membrane by the excessively myristoylated Src disturbed the cell growth.

We then tested the effect of CfrPTP-3 overexpression. The CfrPTP-3-mCherry protein was observed in the whole cytoplasm, but strongly localized both at the nuclei and some unidentified intracellular structures, which in many cases clustered close to a nucleus (Fig. 5C). No particular growth defect was observed compared to the control cells transfected with only the Venus construct. However, the cytoplasm frequently thickened and many small vacuoles, instead of a large single one in the center, appeared in the cytoplasm. Strikingly, the phenotype of CfrSrc was rescued (Fig. 5D) and cells grew as well as the control cells when CfrPTP-3 was co-overexpressed with CfrSrc.

To assess the phenotype of the growth defect statistically, we measured the diameter of each transformant (Fig. 5E). The size of the CfrSrc-overexpressed transformants was significantly smaller than any of the control cells (*p* = 0.0076 by Wilcoxon test with Benjamini-Hochberg correction), the CfrPTP-3-overexpressed cells (*p* = 0.0036), the CfrSrcMut-overexpressed cells (*p* = 0.016), or the cells with both CfrSrc and CfrPTP-3 overexpressed (*p* = 0.014). The experiment was repeated 4 times, and we confirmed that the median sizes of the CfrSrc transformants were consistently smaller than those of the others (Fig. 5F).

It was possible that CfrPTP-3 rescued the growth defect by CfrSrc simply because the ratio of the CfrSrc construct to the whole transfected DNA was reduced. To test this possibility, we cotransfected the CfrSrc construct together with Venus, rather than with CfrPTP-3 (3 μg of Venus and 1 μg of CfrSrc; Fig. 5G middle). The median cell diameter did not differ (*p* = 0.64) from the control experiment (Fig. 5G left). Moreover, simply reducing the amount of the transfected CfrSrc construct to 0.5 μg did not increase the cell size (Fig. 5G right).

In summary, our *in vivo* data indicate that the overexpressed CfrSrc strongly compromises the normal growth of *Creolimax* cell by protein phosphorylation and the co-transfected CfrPTP-3, which suppresses the CfrSrc activity *in vitro*, can rescue the phenotype *in vivo* as well.

## Discussion

Many signaling pathways that control cell growth and differentiation arose prior to the advent of multicellularity. The three-stage lifecycle of ichthyosporeans such as *Creolimax* offers a unique window into the role of tyrosine phosphorylation in cell differentiation. The amoeboid and multinucleate stages show distinct transcriptional profiles^36^. In the multinucleate stage, there is an enhancement of genes associated with cell growth, such as genes involved with DNA replication, translation, and amino acid and RNA metabolism. In the amoeboid stage, there is an upregulation of genes encoding GTPases, kinases, and components of the extracellular matrix. Studies of these components have the potential to reveal new details about the molecular mechanisms of the transition from unicellular to multicellular systems^37–39^.

While the human genome encodes 32 cytoplasmic/non-receptor tyrosine kinases, *Creolimax* CfrSrc is the sole NRTK in the kinome of this organism^18^. CfrSrc is similar to metazoan Src-family kinases with respect to its sequence and domain architecture. CfrSrc possesses tyrosine kinase activity (Fig. 2) and is positively regulated by autophosphorylation (Fig. S3A). SFKs can also autophosphorylate their own C-terminal tail tyrosines, although this process is less efficient than Csk-catalyzed tail phosphorylation^40^. We cannot distinguish activation loop vs. tail phosphorylation in our Western blotting experiments (Fig. S3B); however, the large increase in kinase activity (Fig. S3A) is consistent with activation loop phosphorylation in mammalian SFKs (e.g., in Hck^41^), suggesting that the predominant site for autophosphorylation is within the activation loop.

A notable difference between *Creolimax* CfrSrc and mammalian tyrosine kinases is the identity of the gatekeeper residue in the active site cleft. Resistance to pharmacological ATP-competitive inhibitors is frequently driven by mutation of the gatekeeper residue into a bulkier amino acid such as isoleucine^27^. CfrSrc possesses a leucine residue at the gatekeeper position, and the enzyme is resistant to the inhibitors dasatinib and DSA1 (Fig. 3). Kinase gatekeeper mutations can lead to kinase activation independent of drug binding by stabilizing the hydrophobic spine, a network of hydrophobic interactions that are characteristic of active kinases^42^. The EGFR T790M gatekeeper mutation, which is found in patients with advanced non small cell lung cancer, increases the enzyme’s affinity for ATP^43^. Although the role of CfrSrc in *Creolimax* cells is unclear, its gatekeeper sequence would allow the enzyme to be active, even in an environment with very low ATP or with a naturally-occurring ATP competitor. Interestingly, the living conditions of unicellular holozoans (in particular, whether they are symbiotic or exposed to the natural environment) appear to broadly correlate with the bulkiness of the gatekeeper residue. Similar to *Creolimax*, *S*. *arctica* has only one Src, which has a leucine at the gatekeeper position. *A*. *whisleri* and its close relative *P*. *gemmata* have 5 and 3 Srcs, respectively, which have leucine, valine, isoleucine, and tryptophan and asparagine. *Creolimax*, *S*. *arctica*, *A*. *whisleri*, and *P*. *gemmata* were all discovered in digestive tracts of marine invertebrates. However, *A*. *parasiticum*, which is typically found attached to the exoskeletons of certain arthropods^44^, has a mixed repertoire; 2 out of 4 Srcs have leucine at this position, but the other 2 retain threonine. *C*. *limacisporum*, a free-living unicellular holozoan, has only one Src, which has a threonine residue at this position. To understand why symbiotic ichthyosporeans tend to evolve Src proteins with bulky gatekeeper residues, further experiments would be necessary in conditions reproducing their natural environments.

The regulation of tyrosine phosphorylation appears to play an important role in the normal developmental cycle of *Creolimax*. A study of transcriptional profiles in *Creolimax* showed that the expression levels of CfrSrc are similar in the amoeboid and the multinucleate stages, in contrast to other components of adhesion signaling (e.g., integrins, paxillin, vinculin), which are most highly expressed in the amoeboid stage^36^. This result suggested that CfrSrc is not tightly connected to the adhesion signaling pathway in *Creolimax*. It is possible that as a result of the NRTK reduction that occurred in the ichthyosporean lineage leading to *Creolimax* (and *S*. *arctica*), CfrSrc was decoupled from the regulation of cell adhesion and amoeba motility.

The absence of a Csk kinase in *Creolimax* indicates that CfrSrc must be negatively regulated in a different manner than mammalian c-Src. Other unicellular kinases display weak negative regulation, including *M*. *ovata* Src^30^ and Pak^45^, *M*. *brevicollis* Src^26^ and Abl^46^, and the Src-family kinases from the filastereans *C*. *owczarzaki*^31^ and *M*. *vibrans*^33^. Our data on the specific downregulation of CfrSrc by CfrPTP-3 both *in vitro* (Fig. 4) and *in vivo* (Fig. 5), together with the expression analysis of TKs and PTPs in *Creolimax*, suggest that CfrSrc has low activity during the growing multinucleate stage due to the high dephosphorylation activity of CfrPTP-3. The twofold higher expression of CfrPTP-3 in the amoeboid stage implies an additional function for the further suppression of pTyr signals during this stage. Additional functional analyses focused on the maturation stage, which we did not examine in this study, are necessary.

*Creolimax* has lost the Csk gene itself in the course of evolution, leading to the total disruption of the Src-Csk regulatory machinery. Our results suggest that an existing PTP can be co-opted to take the place of Csk for negative regulation of Src kinase. Previous studies on Src-Csk regulation in unicellular holozoans performed in metazoan cell culture systems demonstrated that Csk is not as tightly connected to the control of Src activity as it is in metazoans. On the other hand, a recent study^47^ showed that Csks from *C*. *limacisporum*, the choanoflagellate *M*. *brevicollis*, and the filasterean *C*. *owczarzaki* can regulate Src in a yeast cell system. The *Creolimax* transformation system described here opens the door to studies of pTyr signaling in intact unicellular eukaryotes under natural conditions. This will ultimately allow an understanding of the roles of tyrosine phosphorylation in the critical developmental transitions in these organisms, and possibly in the putative unicellular ancestor of metazoans.

## Materials and Methods

### Materials

Nickel-nitriloacetic acid resin was purchased from Qiagen. Anti-phosphotyrosine antibody 4G10 was from EMD Millipore. Expression and purification of *M*. *brevicollis* MbSrc1, *M*. *brevicollis* MbSrc4, *C*. *owczarzaki* CoSrc2, and *Yersinia* YOP phosphatase were as described previously^26,31,48^. Human c-Src and inhibitor DSA1 were gifts from Dr. Markus Seeliger (Stony Brook University). Dasatinib was purchased from Selleck Chemicals. The cDNAs for *Creolimax* PTPs CfrPTP-1, CfrPTP-3, CfrPTP-4, CfrPTP-5, and CfrPTP-7 were synthesized by GenScript after optimizing codons for *E*. *coli* expression. Fluorescently labeled pYEEI peptide was synthesized by GeneMed Synthesis, Inc.

### Protein expression and purification

The full-length cDNA for *Creolimax* Src kinase (residues 1–586) was amplified by PCR and cloned into the BamHI and NotI sites of plasmid pFastbac-Htb (Thermo Fisher Scientific). Recombinant baculovirus was produced using the Bac-to-Bac system (Thermo Fisher). The amplified virus was used to infect a 600 ml spinner culture of *Spodoptera frugiperda* (Sf9) cells. After 72 hours, cells were lysed in a French pressure cell. His-tagged CfrSrc was purified on 4 mL of nickel-nitrilotriacetic acid resin, using methods described previously^26^, with the following modification: the enzyme was eluted from the column using steps of 25, 50, 75, and 100 mM imidazole. Purified CfrSrc was stored at −80 °C.

DNA encoding the CfrSrc SH2 domain (residues 152–255) was amplified by PCR and cloned into the BamHI and EcoRI sites of plasmid pGEX-4T-1 (GE Healthcare). The GST-SH2 fusion protein was expressed in 1 L *E*. *coli* BL21(DE3) cell cultures. Cells were lysed in a French pressure cell, and the SH2 domain was purified on glutathione-agarose resin (Molecular Probes)^26^. GST-SH2 protein was eluted with 20 mM glutathione in 50 mM Tris (pH 8.0), concentrated on an Ultracel 10 kDa concentrator (Amicon), and stored at −80 °C.

The cDNAs for *Creolimax* PTPs were synthesized commercially and were subcloned into the BamHI and EcoRI sites of plasmid pGEX-4T-1. The constructs included the following residues: CfrPTP-1, residues 1–531; CfrPTP-3, residues 12–289; CfrPTP-4, residues 1–284; CfrPTP-5, residues 1–443; CfrPTP-7, residues 128–542. The proteins were expressed in *E*. *coli* BL21(DE3) cells and purified on glutathione-agarose resin, as described above. For some experiments, the phosphatases remained immobilized on the resin and were stored at 4 °C.

### Enzyme assays

Two kinase assays were used: (1) Kinetic experiments were performed with a continuous spectrophotometric assay^49^. For the determination of K_m_(ATP), the enzyme concentration was 400 nM. (2) Kinase activity was also measured using phosphocellulose paper binding assays with [γ-^32^P]ATP^50^. Reactions contained 20 mM Tris-HCl (pH 7.4), 10 mM MgCl_2_, 0.25 mM ATP, with varying concentrations of peptide substrates and 100–500 cpm/pmol of [γ-^32^P]ATP. The following peptides were used: Src peptide, AEEEEIYGEFEAKKKKG; EGFR peptide, AEEEEYFELVAKKKG; IR peptide, KKEEEEYMMMM; Abl peptide, EAIYAAPFAKKKG; IRS-1 peptide, KKSRGDYMTMQIG; and Kemptide, LRRASLG^51–53^. For experiments comparing different peptides, the concentration of CfrSrc was 800 nM and the peptide concentration was 500 μM. To examine the effects of *M*. *brevicollis* MbCsk or human Csk on the activity of CfrSrc, the enzymes were preincubated for 15 minutes at 30 °C prior to assay.

The effects of *Creolimax* tyrosine phosphatases on CfrSrc were measured in two ways. To measure CfrSrc kinase activity, the immobilized phosphatases (2 µg) were incubated with CfrSrc (5 µg) for 30 minutes at room temperature, and were removed by centrifugation. CfrSrc activity was then tested using the phosphocellulose paper binding assay. To measure effects on CfrSrc autophosphorylation, the reactions were analyzed by SDS-PAGE with anti-phosphotyrosine Western blotting.

### Fluorescence polarization measurements

The fluorescent pYEEI peptide (5-carboxyfluorescein-GEPQpYEEIPI) was purified by semipreparative HPLC on a Vydac C18 column. The peptide was lyophilized and resuspended in a 1:1 mixture of water: DMSO. Fluorescence polarization was measured with final peptide concentrations of 0.25 μM or 0.5 μM in 50 mM Tris (pH 7.4) using a Victor2 instrument (Wallac). The data were analyzed with Origin software (Originlab, v. 7).

### Culture of Creolimax

A live culture of *Creolimax*, originally provided by Wyth Marshall (BC Centre for Aquatic Health Sciences, Campbell River, Canada) was maintained at 12 °C in the Difco Marine Broth (BD).

### DNA constructs for expression in Creolimax

The overexpression vectors were constructed as in^4^, with pCR2.1 (Thermo) as the backbone. Vectors were made with or without a mCherry tag at the C-terminus. We confirmed that the presence of the fluorescent protein does not significantly alter the phenotype of transformants.

### Electroporation

The *Creolimax* cells were grown detached from the bottom of culture flask by rotational shaking at 70 rpm. The culture of 3 days to 2 weeks old was filtered with 10 μm mesh to collect only small cells (amoebae or uninucleate cells). Using Neon (Thermo), we applied to the cells 7 pulses of 2,500 V for 3 ms in the 10 μL electrode tip filled with R buffer (Thermo), followed by 5 pulses of 500 V for 50 ms, and immediately transferred them into the Marine Broth. The cells were kept on ice for 1–2 hours and incubated at 12 °C for 48 hours before the observation. The confocal microscope FV1000 (Olympus) and the fluorescent microscope CX41 (Olympus) were used for the observation. The images were processed with the Fiji program (https://fiji.sc/). The diameters of cells were calculated from the perimeters measured by Fiji.

### Phylogenetic tree analysis

Amino acid sequence data were retrieved from GenBank and the proteome databases of *Capsaspora*^5^ and *Creolimax*^36,54^. The published alignments^18,55^ were manually modified and used for the tree inferences. Phylogenetic trees were inferred from the whole sequence (SH3, SH2, and kinase domains) of TK (364 amino acid positions), and the catalytic domain sequence of PTP (192 amino acid positions) by the Maximum Likelihood (ML) method, using the LG-Γ model implemented in RAxML v7.2.8^56^.

### Expression quantification by RNAseq

The RNAseq data published previously^36^ were analyzed by Tophat2 and Cufflinks2 programs^57^. The fragments per kilobase of exon per million mapped fragments (FPKM) values were calculated from three independent samples. The significance of stage-specific expression difference was evaluated by the comparison between p-value and false-discovery rate (FDR) after Benjamini-Hochberg correction.

### Data availability

All data generated or analyzed during this study are included in this published article (and its Supplementary Information files).

## Electronic supplementary material

Supplemental figures
