# Supplementary material for: Src signaling in a low-complexity unicellular kinome
Source: Sci Rep. 2018 Mar 29;8:5362. doi: 10.1038/s41598-018-23721-8 (PMC5876402; doi:10.1038/s41598-018-23721-8)
Supplement: Supplementary file 1 — Supplemental figures [file 41598_2018_23721_MOESM1_ESM.pdf]

Supplemental figures

H. Suga and W. T. Miller, "Src signaling in a low-complexity unicellular kinome"

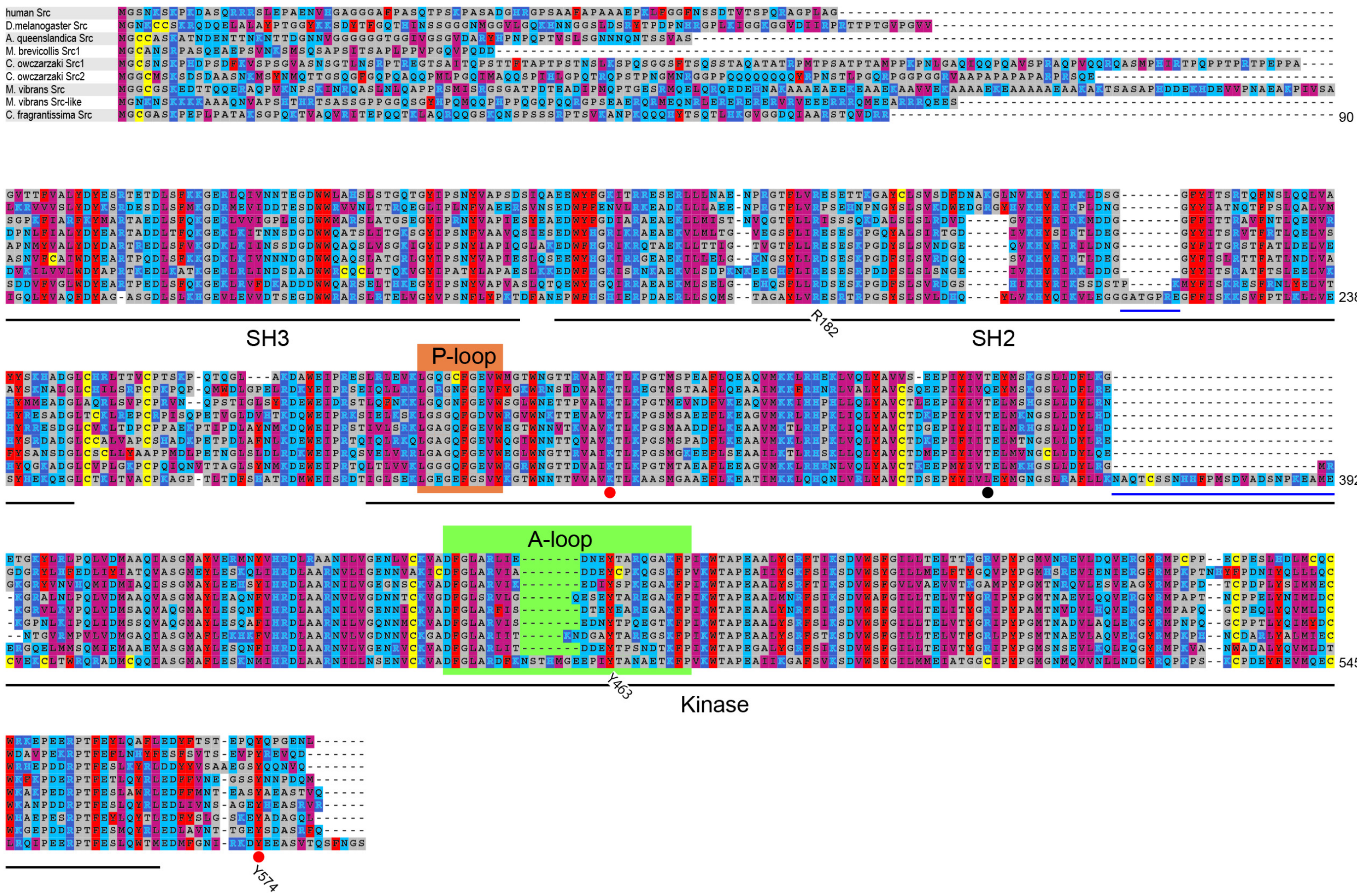

Supplemental Figure 1

Amino acid alignment of Src proteins. The N-terminal sequences prior to the SH3 domain are unaligned. SH3 domain, SH2 domain, and kinase domain are underlined, according to the SMART (http://smart.embl-heidelberg.de) profiles. Amino acids are numbered by the *Creolimax* sequence. The gatekeeper Thr residue is labeled by a filled circle. The *Creolimax*–specific insertions in the SH2 domain and near the hinge region are highlighted by blue lines. The P-loop and A-loop of catalytic domain is shaded orange and green, respectively, according to Seeliger *et al.*[1]. The mutated amino acids in the CfrSrcMut construct are indicated by red circles. Amino acids are colored by their physicochemical properties.

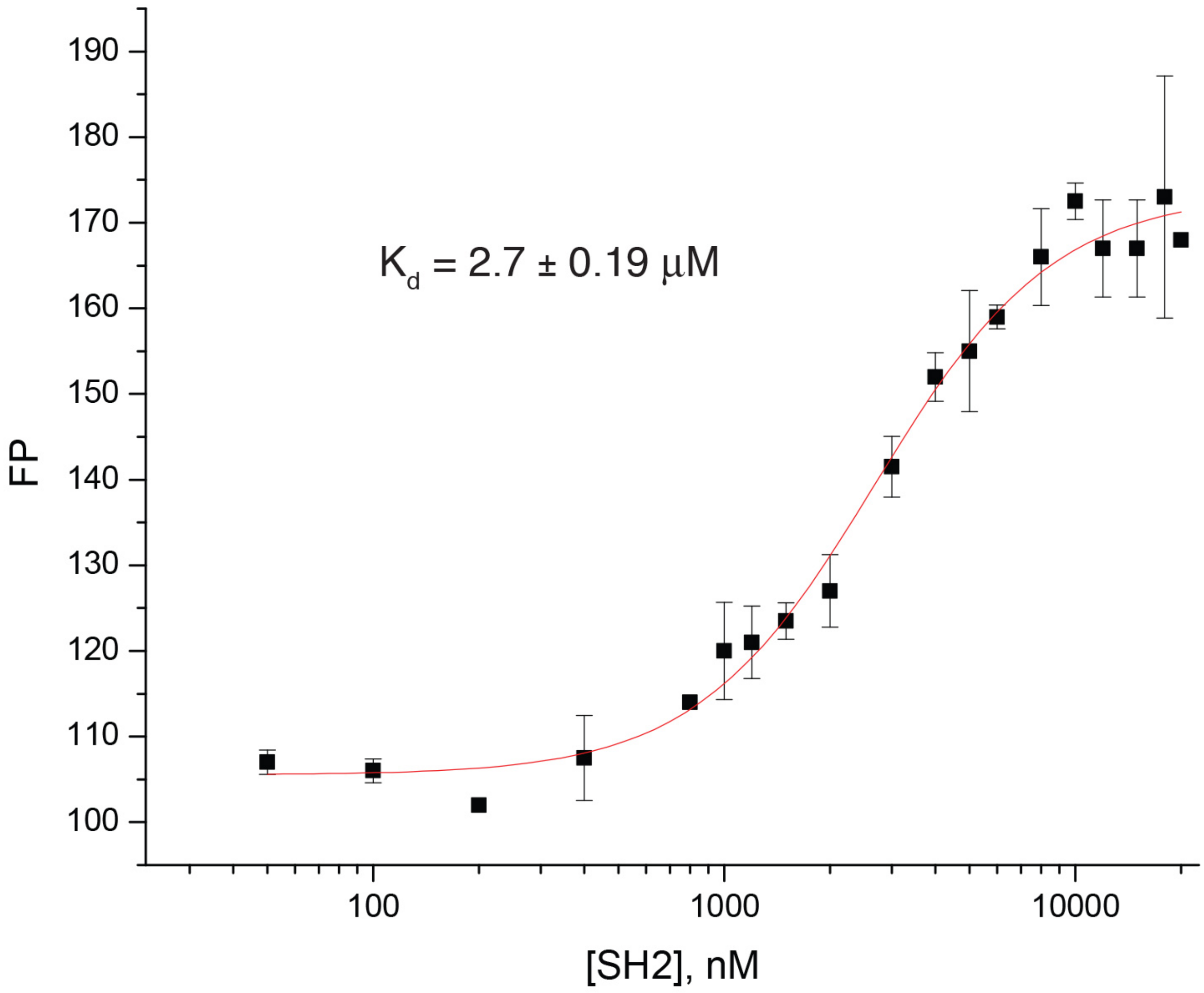

Supplemental Figure 2

Binding of a pTyr-containing peptide to the CfrSrc SH2 domain was measured by fluorescence polarization. The red line shows curve fitting. Three independent measurements gave K_d values of 2.7 ± 0.19 μM, 2.9 ± 0.25 μM, and 4.0 ± 0.48 μM.

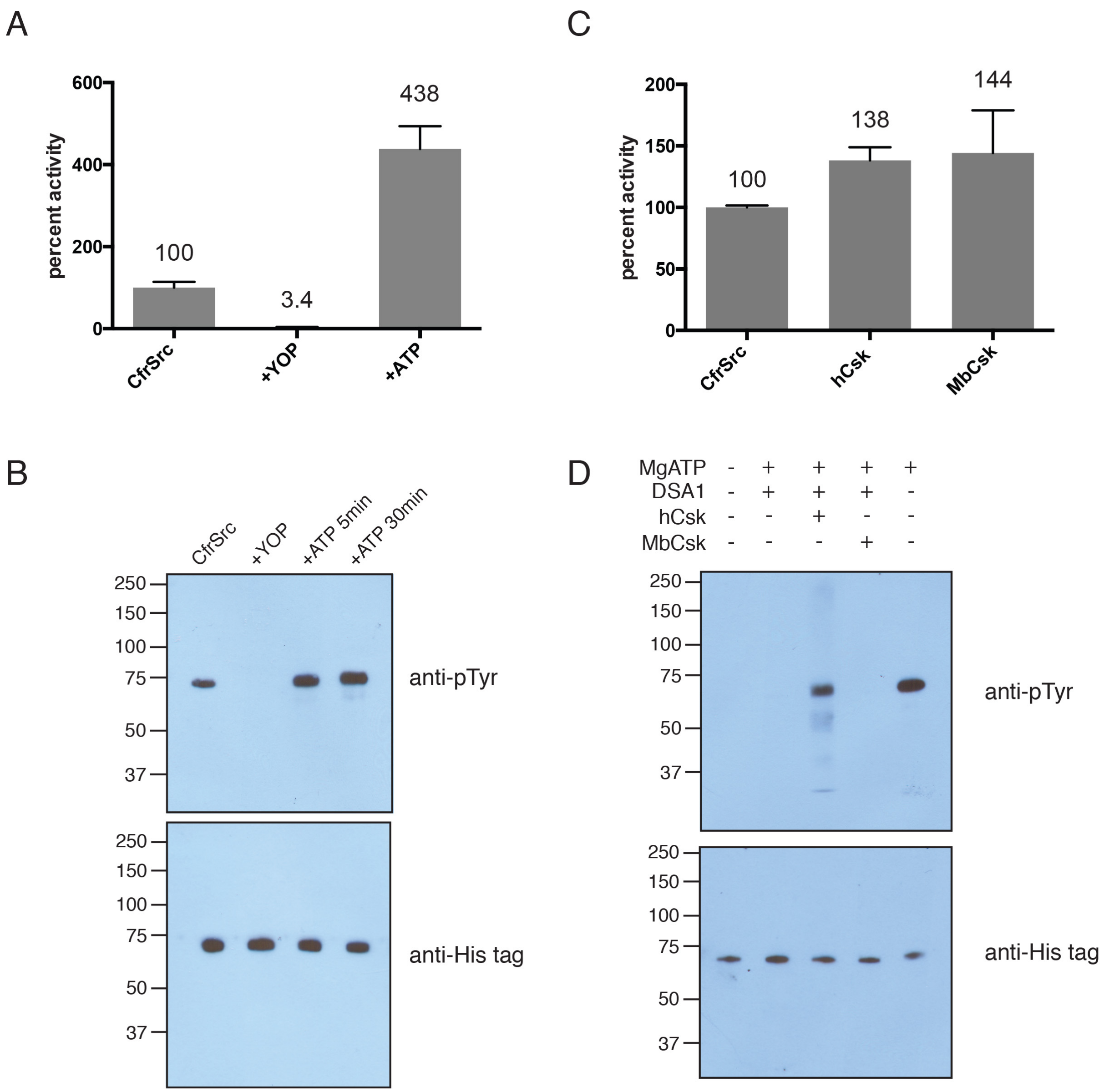

Supplemental Figure 3

Regulation of CfrSrc. (A) The activity of purified CfrSrc (1 μM) was measured directly, or after treatment with immobilized YOP phosphatase, or after treatment with YOP and subsequent incubation with 1mM ATP. Activity toward the Src peptide was measured with the phosphocellulose binding assay using [γ-^{32}P]ATP. (B) CfrSrc was analyzed by SDS-PAGE and Western blotting with anti-pTyr and anti-His tag antibodies. The enzyme was measured directly, after treatment with immobilized YOP, and after treatment with YOP and subsequent incubation

with 1mM ATP (5 and 30 minute reactions). (C) CfrSrc activity was measured after treatment with immobilized human Csk (hCsk) or *M. brevicollis* Csk (MbCsk). (D) Phosphorylation reactions with CfrSrc L352T were analyzed by SDS-PAGE and Western blotting with anti-pTyr and anti-His tag antibodies. L352T was first dephosphorylated by treatment with YOP. Phosphorylation reactions with L352T (15 minutes at 30°C) were carried out in the presence or absence of 1mM ATP/$MgCl_2$, 5μM DSA1, 2μM soluble hCsk, or 2μM soluble MbCsk, as indicated by the symbols above the gels. The right-hand lane shows an L352T autophosphorylation reaction in the absence of DSA1.

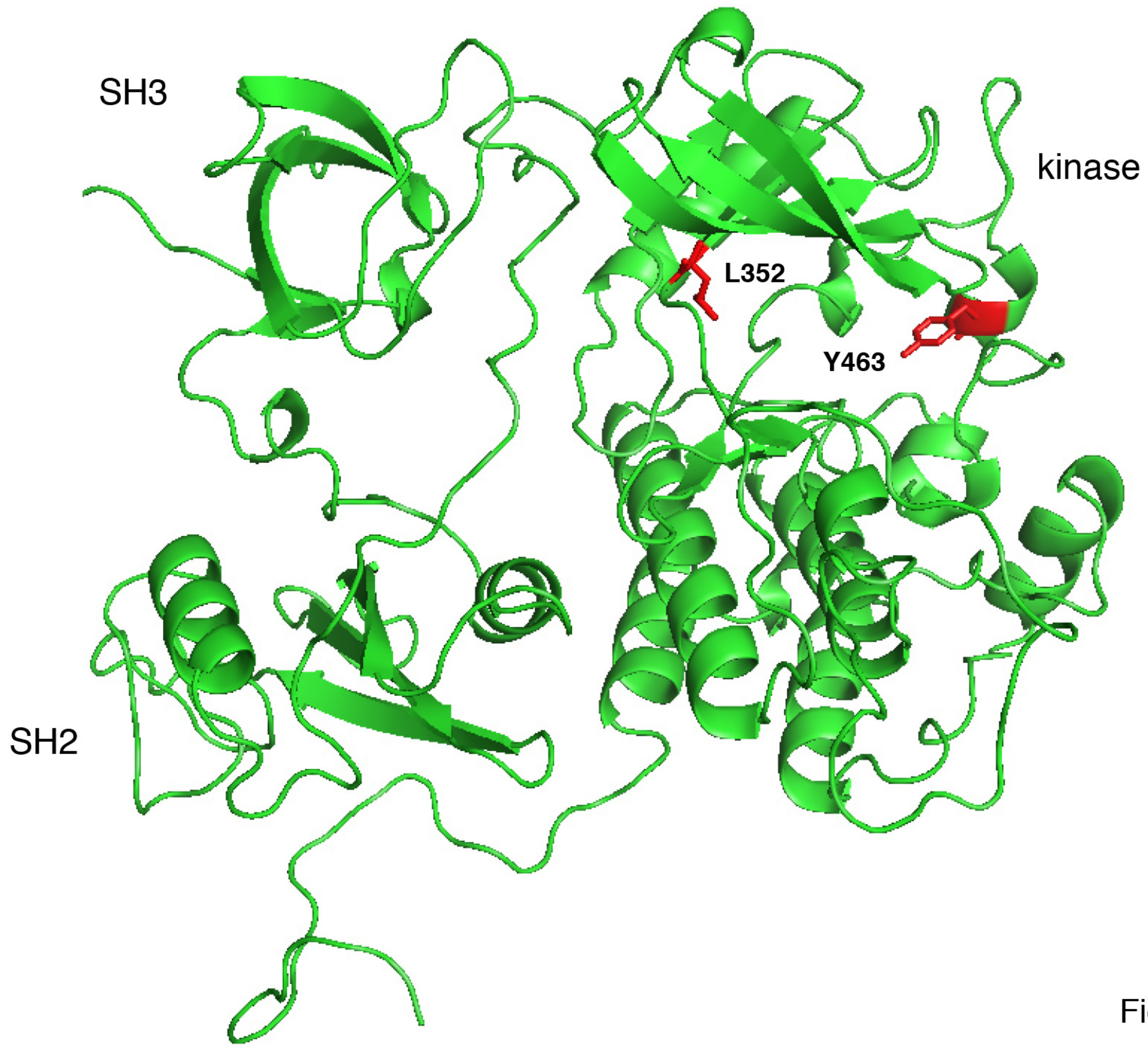

Fig. S4

Supplemental Figure 4

Structural model of CfrSrc. The HHPred software package[2] was used to identify related protein sequences, and the three-dimensional structure of CfrSrc was modeled on that of Src kinase in a complex with a quinazoline inhibitor (PDB code 2H8H) using MODELLER. The positions of L352 and Y463 are shown in red.

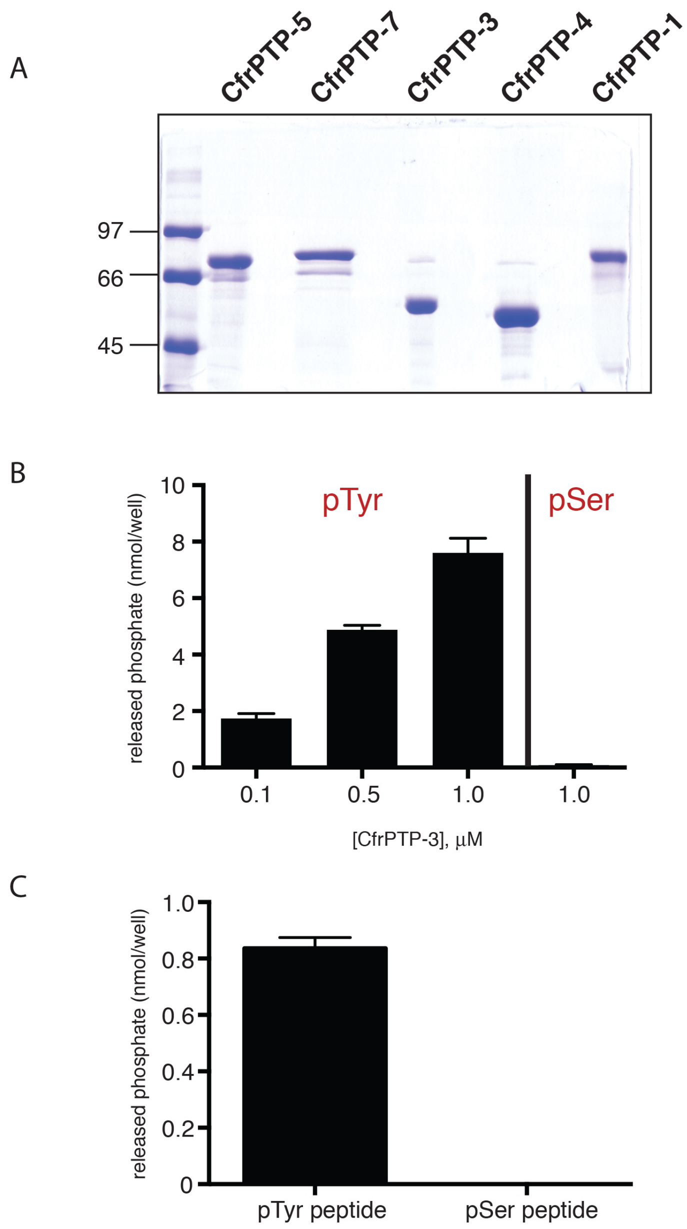

Supplemental Figure 5

(A) Purification of CfrPTPs. GST-tagged CfrPTPs were expressed in *E. coli* and purified by glutathione agarose chromatography. The purified proteins were analyzed by SDS-PAGE and Coomassie staining. The full-length gel is shown. (B) The activity of CfrPTP-3 at the indicated concentrations was measured towards pTyr or pSer (60μM) using a malachite green phosphate assay kit (abcam). (C) The activity of 0.5 μM CfrPTP-3 toward a pTyr-containing peptide (QSFASDPKpYATPQVI, 30 μM) or a pSer-containing peptide (LRRApSLG, 60μM) was measured using the malachite green assay kit.

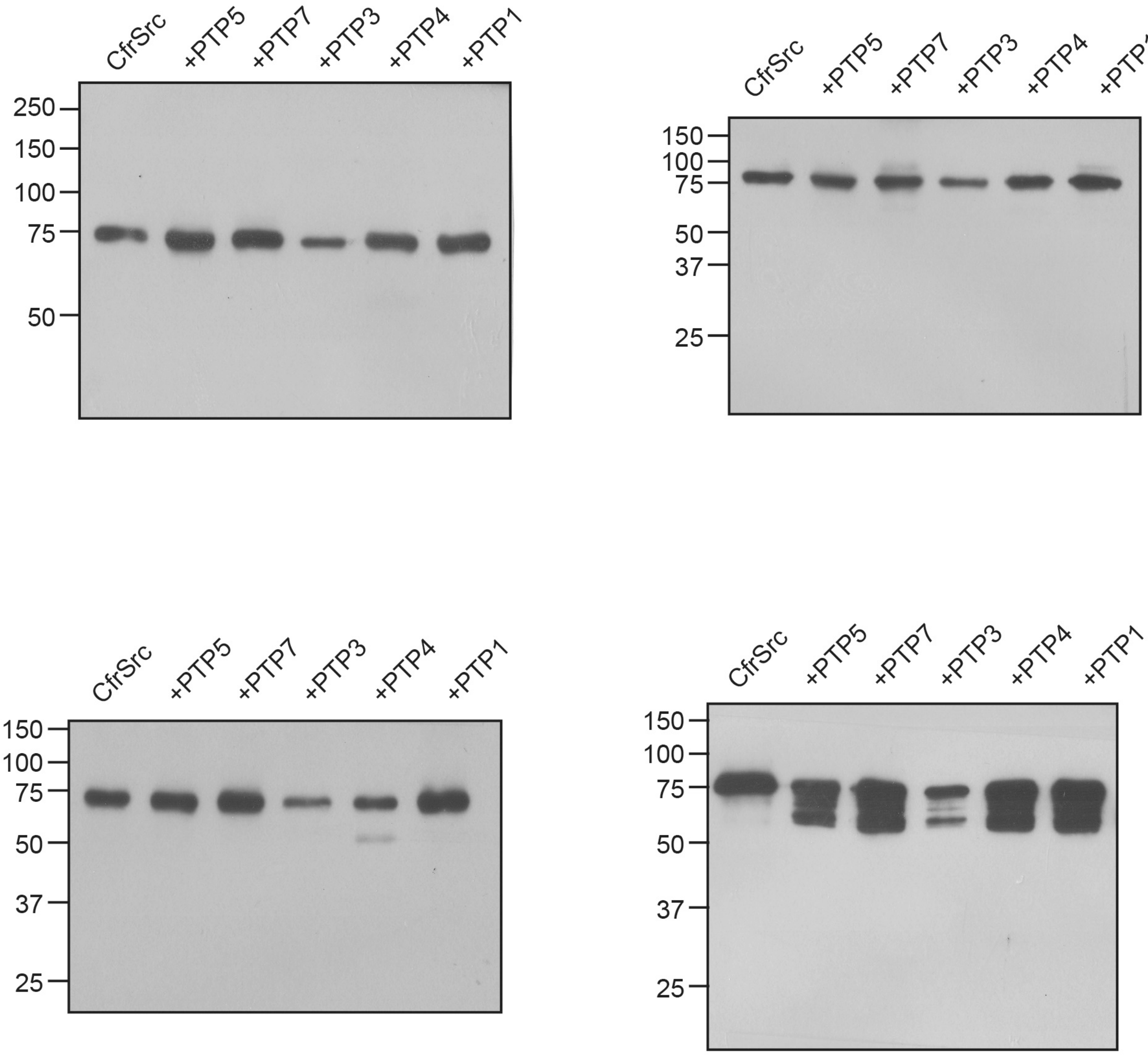

Supplemental Figure 6

Full-length blots showing the 4 independent experiments used for Figure 4E.

Supplemental references

1 Seeliger, M. A. *et al.* c-Src binds to the cancer drug imatinib with an inactive Abl/c-Kit conformation and a distributed thermodynamic penalty. *Structure* **15**, 299-311 (2007).

2 Alva, V., Nam, S. Z., Soding, J. & Lupas, A. N. The MPI bioinformatics Toolkit as an integrative platform for advanced protein sequence and structure analysis. *Nucleic Acids Res* **44**, W410-415, doi:10.1093/nar/gkw348 (2016).
